# Higher readability of institutional websites drives the correct fruition of the abortion pathway: A cross-sectional study

**DOI:** 10.1371/journal.pone.0277342

**Published:** 2022-11-04

**Authors:** Amerigo Ferrari, Luca Pirrotta, Manila Bonciani, Giulia Venturi, Milena Vainieri

**Affiliations:** 1 Institute of Management, MeS (Management and Health) Laboratory, Sant’Anna School of Advanced Studies, Pisa, Tuscany, Italy; 2 Institute of Computational Linguistics “A. Zampolli” (ILC-CNR), Italian Natural Language Processing Laboratory (ItaliaNLP Lab), National Research Council, Pisa, Tuscany, Italy; Universita degli Studi della Campania Luigi Vanvitelli, ITALY

## Abstract

**Background:**

In Italy, abortion services are public: therefore, health Institutions should provide clear and easily readable web-based information. We aimed to 1) assess variation in abortion services utilisation; 2) analyse the readability of institutional websites informing on induced abortion; 3) explore whether easier-to-read institutional websites influenced the correct fruition of abortion services.

**Methods:**

We identified from the 2021 administrative databases of Tuscany all women having an abortion, and–among them–women having an abortion with the certification provided by family counselling centres, following the pathway established by law. We assessed variation in total and certified abortion rates by computing the Systematic Component of Variation. We analysed the readability of the Tuscan health authorities’ websites using the readability assessment tool READ-IT. We explored how institutional website readability influenced the odds of having certified abortions by running multilevel logistic models, considering health authorities as the highest-level variables.

**Results:**

We observed high variation in the correct utilization of the abortion pathway in terms of certified abortion rates. The READ-IT scores showed that the most readable text was from the Florence Teaching Hospital website. Multilevel models revealed that higher READ-IT scores, corresponding to more difficult texts, resulted in lower odds of certified abortions.

**Conclusions:**

Large variation in the proper fruition of abortion pathways occurs in Tuscany, and such variation may depend on readability of institutional websites informing on induced abortion. Therefore, health Institutions should monitor and improve the readability of their websites to ensure proper and more equitable access to abortion.

## Introduction

The ambivalent Italian law 194/1978 regulates abortion but claims to refuse abortion as a means of birth control, recognizing motherhood as a social value to preserve [1]. In Italy, abortion can be performed within the first 90 days of pregnancy, after which the need to protect the foetus’s life overcomes the right of women to terminate the pregnancy, except for life-threatening events or foetal diseases. Women aiming to abort must visit free family counselling centres [2] to obtain a certificate attesting that they are pregnant and wish to have an abortion. Then, they must wait a week before terminating the pregnancy, and after the abortion, they are advised to visit back the family counselling centre to receive psychological support and contraception [3]. The law declares that abortion is a right to be ensured freely to all women, but at the same time offers health professionals the chance to conscientious objection [4, 5].

Abortion rates in Italy have been declining during the last decades, reaching values below many European countries (5.8 per 1,000 women in 2019) [6]. However, wide variation occurred among the Italian Regions (from 4.1 to 7.9), mainly due to difficult access to abortion services in some Regions (especially in the South) [7]. Determinants of such a variation may be the scarcity of family counselling centres locally, the excess of conscientious objectors, or deficient communication strategies. As for Tuscany, several performance indicators tracking abortion rates and access to abortion are available in the Performance Evaluation System of Sant’Anna School of Advanced Studies [8, 9]. However, studies investigating the determinants of greater or lesser access to abortion services are lacking.

Considering the widespread dissemination of health-related information, the ability to understand such information can influence the degree and fairness of access to health services. Therefore, the concept of health literacy is nowadays more important than ever. Health literacy is defined as the ability to process and understand health-related information to be able to take decisions independently [10]. Low health literacy implies less ability to interpret health-related messages and consequently a poor and incorrect utilisation of health services [11]. Italy registered an inadequate health literacy on more than half of the population [12]. The quality of online health information has already been explored in the Italian context, demonstrating that a low clarity of information can undermine the efforts in effectively informing and involving the public through health services [13].

As for abortion services, some studies have explored the readability of web-based sources informing women about the abortion pathway [14, 15], but no data is available in the Italian context. The importance of pre-abortion communication strategies for an informed, conscious, and shared decision has been described [16–18]. Particularly, the Web has become the main source for health-related information, also on induced abortion [19, 20]. This is especially true for people with a low education level [21]. Indeed, less educated women are more likely to have an abortion, as abortion rates among non-graduated women in Italy were at least 3-fold higher than among graduated ones in 2019 [7]. Therefore, the readability (in terms of linguistic complexity) of online sources informing women on the abortion pathway is a major issue to be addressed, as differences in understanding and interpreting such information may affect the equity of access to abortion services or the correct fruition of the pathway [22].

We hypothesized that wide intra-regional variation in access to abortion services existed among the health districts of Tuscany and that the quality and readability of web information on the abortion pathway provided by Tuscan Local Health Authorities (LHAs) and Teaching Hospitals (THs) might influence the correct fruition of abortion services locally. Therefore, we aimed to

1. Assess practice variation in the access and proper fruition of abortion services among Tuscan health districts
2. Quantify the readability of institutional websites informing women how to properly access abortion services
3. Explore whether easier-to-read institutional websites might influence the correct use of abortion services

## Methods

### Setting, study design, and data source

The Italian National Health Service follows a decentralized Beveridge-like model. Regions are responsible for the organization and delivery of health services within their territories, and they are provided with political, administrative, fiscal, and legislative autonomy [23]. The regional healthcare system of Tuscany receives around 6% of the healthcare fund, providing health services to 3.7 million inhabitants. It is structured in three Teaching Hospitals (THs) and three Local Health Authorities (LHAs), financed by a capitation-weighted budget. The three THs (Pisa, Siena, Florence) are considered as autonomous health authorities lacking territorial services, while the three LHAs (North-West, Centre, South-East) comprise both hospital and territorial facilities and are divided into twenty-six health districts. More than 95% of hospitals are public. The last national assessment by the Ministry of Health reported Tuscany as one of the most performing Italian Region.

In this retrospective cross-sectional study, we used two different sources: 1) 2021 health administrative databases from Tuscany, and 2) LHA and TH webpages informing women on the abortion pathway. As an observational study, it has been reported according to the STROBE checklist.

Regional administrative data are provided to our laboratory thanks to a collaboration agreement with the Regional Health Service of Tuscany. In this study, we used the 2021 Voluntary Termination of Pregnancy (VTP) Database, comprising all women that had an abortion in 2021, regardless of the clinical setting. We also employed the 2021 Aggregated Population Database of Tuscany to obtain information on the number of women residing in each district. The Regional Health Information Office of Tuscany routinely checks the data quality and anonymize data by assigning to each patient an encrypted unique identifier. This identifier is the same for all administrative claims and prevents the patient’s personal data and sensitive information from being traced back, in compliance with the European GDPR 2016/679 and Italian law on privacy 101/2018. The use of individually collected and anonymized administrative data for research activities without the need to obtain informed consent and approval from the ethics committee has been authorized by the Italian Data Protection Authority in 2012 [24].

Furthermore, institutional webpages informing women on how to access the abortion pathway across health districts were identified from the LHA and TH official websites. The search was performed in January 2022 as data collected from regional administrative databases referred to the previous year (2021). The websites of the three LHAs and the three THs were screened independently by the first and the second authors. Then, both the first and second authors accessed and revised together the selected websites to identify and copy the text-based content on induced abortion. We assumed that each patient had looked for information on the abortion pathway on the website of the health authority (TH or LHA) where she had the abortion.

### Population

We identified from the VTP Database:

1. The entire population of women (aged 14 to 49) having an induced abortion in Tuscany in 2021
2. The subgroup of women having an induced abortion with the certificate provided by free family counselling centres (using the dichotomous variable “certification”).

### Variation in abortion rates

We computed the numbers of total abortions and certified abortions for each health district (n = 26) and expressed them as rates per 1,000 women, dividing the numerators by the female population of each health district, and multiplying them by 1,000. Total abortion rates were also calculated among non-Italian and under-25 women separately (modifying the denominators accordingly).

Variation among health districts was assessed by computing at the regional level the Systematic Component of Variation (SCV), which represents the systematic variation considered to be beyond chance [25, 26]. Considering I as the number of the health district (n = 26) in Tuscany, y_i_ as the observed numerosity, and e_i_ as the expected number of observations, the SCV was calculated as follows:

$$SCV=\frac{1}{I}(\sum \frac{{\left(y_{i}-e_{i}\right)}^{2}}{e_{i}^{2}}-\sum \frac{1}{e_{i}})$$

### Institutional website readability

We obtained six texts from institutional websites and analysed them with the READ-IT tool [13, 27], which is the first Automatic Readability Assessment Software available for the Italian language, based on Natural Language Processing and Machine Learning techniques (S2 Table) [http://www.ilc.cnr.it/dylanlab/apps/texttools/?tt_user=guest]. According to the set of several linguistic features, the READ-IT tool provides four different scores (Base, Lexical, Syntactic, Global) of linguistic complexities ranging between 0 and 100: the easier is a text/sentence, the lower the score.

Specifically, the Base model considers only traditional raw characteristics of text (i.e., sentence and word length); the Lexical model includes a combination of raw and lexical features (e.g., the internal composition of the vocabulary of the analysed text with respect to the Basic Italian Vocabulary); the Syntactic Model is a combination of the previous features in addition to morpho-syntactic (e.g., distribution of Parts-Of-Speech such as nouns, verbs, adjectives, etc.) and syntactic characteristics of text (e.g., the structure of verbal predicates and subordinates, the order of words, etc.); and the Global score is a combination of all features [28].

The tool was successful tested in several application scenarios [29], also including the physician-patient communication of health–related information [21, 23]. For the specific purpose of this study, the readability scores of the text of each website was computed as the average of the scores obtained for each sentence of the text. The reason is that readability indexes are highly correlated with document lengths and the considered websites are characterised by different lengths in terms of sentences.

#### Influence of institutional text readability on the proper fruition of the abortion pathway

We considered the entire population of women having an abortion in 2021 in Tuscany and using the dichotomous variable “certification” collected for each woman we identify women having certified abortions. We built four multilevel logistic models on Stata Software to explore how each READ-IT score (Base, Lexical, Syntactic, Global) influenced the odds of certified abortion. While the dataset was organized at the individual level, the READ-IT scores were computed for the six websites of the health authorities (THs or LHAs) providing abortion services. Therefore, the scores were attributed to each woman based on the hospital where the abortion was provided, depending on whether it was a Teaching Hospital or a Local Health Authority hospital. In fact, the provider hospital (n = 26) was taken as the second-level grouping variable. Finally, we included in the models several woman-related covariates, which are shown in detail in S1 Table.

## Results

In 2021, 3,824 fertile women (aged 14 to 49 years) residing in Tuscany had an abortion in 26 Tuscan hospitals. Among them, 2,054 women (53.7% of the total) had obtained from a counselling centre the certificate attesting their willingness to abort, as required by law 194/1978 [1]. Surgical abortions (40.4%) were performed less frequently than medical ones (59.6%). 93.6% of abortions occurred within the first 90 days of pregnancy. The median waiting times from the request to the performance of the abortion were 6 days. Considering the population of Tuscan women aged 14 to 49 years (n = 736,064), overall abortion rates were 5.2 per 1,000 women, while certified abortion rates 2.8 per 1,000 women. Please, see Table 1 for further information on the sociodemographic features of our study population.

**Table 1:** Sociodemographic characteristics of women.

Women (n = 3,824)	
**Age**, mean (± SD)	31.6 (± 7.3)
**Age groups**, % (n)	
Under 18 years	2.6 (98)
18 years or more	97.4 (3,726)
25 years or less	23.3 (890)
**Gestational age,** % (n)	
First 90 days of pregnancy	93.6 (3,565)
Beyond the first 90 days	6.4 (245)
Missing	14
**Nationality**, % (n)	
Italian	64.6 (2,469)
non-Italian	35.4 (1,355)
**Educational level**, % (n)	
Low education (elementary or middle school)	72.3 (2,764)
High education (high school or university)	27.7 (1,060)
**Employment status,** % (n)	
Employed	52.1 (1,878)
Unemployed	38.3 (1,381)
Students	9.6 (348)
Missing	217
**Marital status,** % (n)	
Single	60.0 (2,043)
Married	30.3 (1,030)
Other	9.7 (329)
Missing	422
**Type of abortion**, % (n)	
Medical	59.6 (2,168)
Surgical	40.4 (1,467)
Other	189
**Previous children,** median (IQR)	1.0 (2.0)
**Previous induced abortions,** median (IQR)	0.0 (1.0)
**Waiting times**, median (IQR)	6.0 (8.0)

n: number; SD: standard deviation; IQR: interquartile range.

### Variation in abortion rates

**Fig 1:**
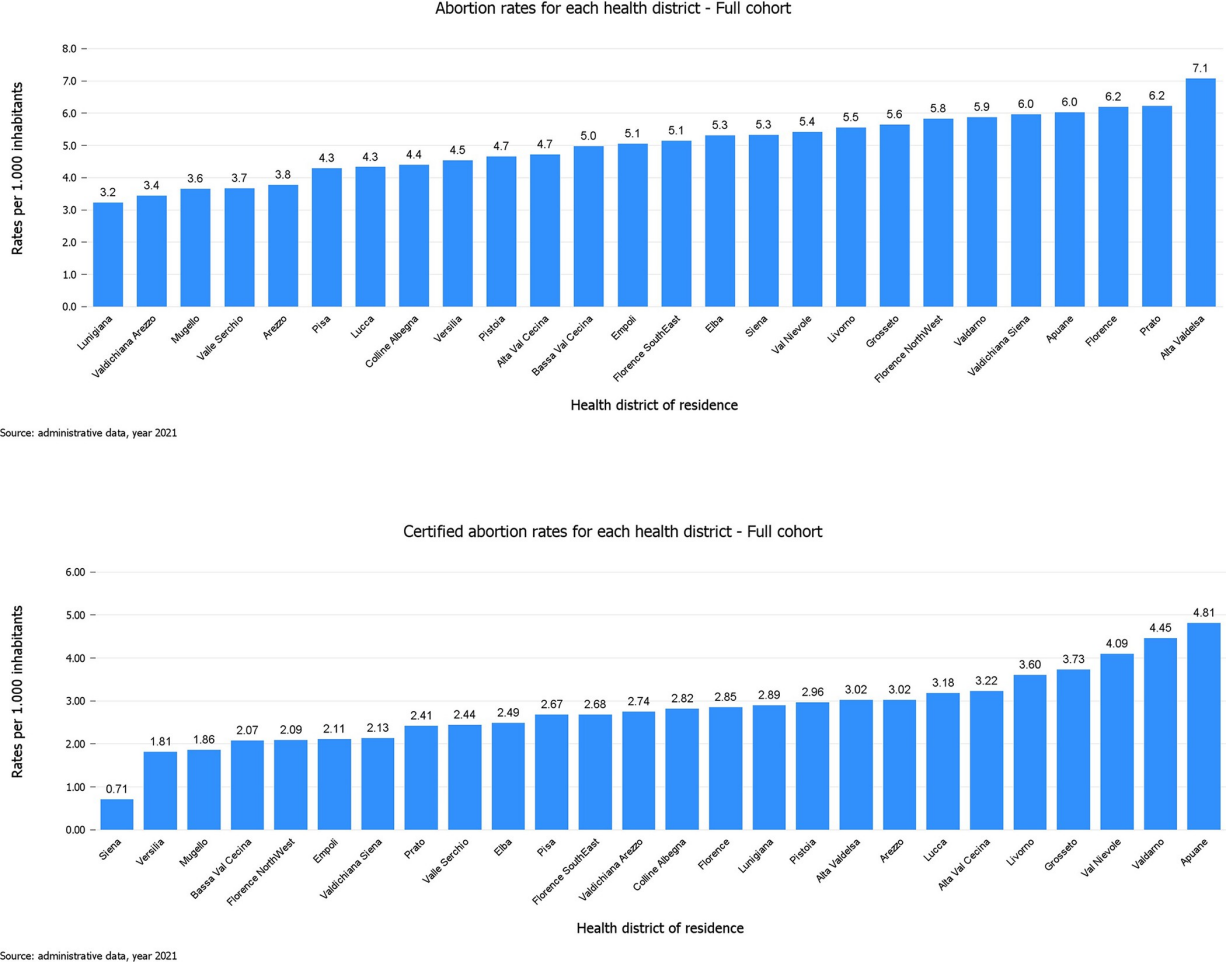
Total and certified abortion rates per 1,000 women.

We observed low variation in abortion rates (Fig 1), as we found 2.2-fold difference between the lowest-rate district and the highest-rate district (from 3.2 to 7.1 per 1,000). After calculating standardized abortion rates adjusted for age, we found an SCV of 2.4% (Table 2), meaning that 2.4% of variation in abortion rates among health districts exceeds random variation, thus being systematic. According to the literature, an SCV below 3.0% indicates low variation among health districts [30].

**Table 2:** Systematic components of variation. According to the literature, an SCV < 3.0% indicates low variation, an SCV between 3.0% and 5.4% moderate variation, and an SCV > 5.4% high variation [30].

Rates	SCV	Interpretation
Total abortions	2.4%	Low variation
Total abortions among under-25 women	2.2%	Low variation
Total abortions among non-Italian women	2.5%	Low variation
Certified abortions	7.5%	High variation

The Systematic Component of Variation (SCV) was expressed as a percentage. The SCV is a method of standardization in which age grouping (five-year age grouping in our study) is assumed to change the risk of abortion by a fixed multiplicative factor. The SCV estimates the relative systematic component of variation between health districts by subtracting the random component of variance (variance within districts) from the estimate of the total variance. Therefore, the SCV represents the systematic variation considered to be beyond chance, thus being systematic.

We also stratified abortion rates by age and nationality, modifying the denominator accordingly (S1 Fig). In particular, we computed abortion rates among under-25 women as they have been involved–at the end of 2018 –in a regional campaign providing them with all contraceptive methods free of charge after medical evaluation at family counselling centres [31]. Abortion rates among under-25 women varied from 1.9 to 6.4 per 1,000 women, and the SCV was 2.2% (Table 2), suggesting low variation [30]. Finally, abortion rates among non-Italian women were generally higher than in the full study population (from 4.5 to 17.1 per 1,000 women) but they had the same variation level (low, SCV = 2.5%) (Table 2).

As for certified abortion rates (Fig 1), we observed a 6.8-fold variation between the lowest-rate district and the highest-rate district (from 0.7 to 4.8 per 1,000). The SCV was 7.5% (Table 2), indicating high variation in certified abortion rates among health districts [30].

### Institutional website readability

The Global READ-It scores–summarizing the other three READ-IT scores–ranged from 35.0 to 73.7 thus showing that the websites were characterised by a medium-low level of readability (S2 Table). Specifically, the most readable text informing women on how to properly access the abortion pathway was obtained from the Florence TH website, while the most difficult-to-read text was from the Centre LHA webpage. Interestingly, the rankings of all websites based on READ-IT scores were very similar when considering and comparing Base, Syntactic and Global scores. In particular, the Florence TH website was always found to be the most readable one. In contrast, Centre LHA readability scores were always the highest (i.e., worst), except for the Base READ-IT score which was higher for the Pisa TH website–as it contained longer sentences.

It is worth noting that the order of the considered texts was slightly different when focusing on Lexical READ-IT scores. In this case, the easiest site to read was the Siena TH one (with a score of 59.5), while the most difficult text remained the Centre LHA one (69.4). Indeed, the Siena TH website contained the highest percentage of vocabulary belonging to the Basic Italian Vocabulary (86.1%), while the Centre LHA website had the lowest distribution of simple words (67.7%). Finally, we observed that the Lexical READ-IT scores obtained from all websites were on average higher than the other READ-IT scores. One possible explanation is that the readability of the websites is more negatively affected by technical jargon or domain-specific terminology than by the morpho-syntactic and syntactic features of the texts.

### Influence of text readability on the proper abortion fruition

While for the first research question we expressed certified (and total) abortions as rates computed at the population level for each health district, for this research question we considered the entire population of women having an abortion in Tuscany in 2021, and–among them–we identified women having a certified abortion using the relative dichotomous variable, as previously described. More detailed data on certified abortions are available in S3 Table.

As shown in Table 3, we found that lower odds of having a certified abortion were driven by higher Lexical (OR = 0.94, p = .017), Syntactic (OR = 0.95, p = .025), Base (OR = 0.91, p = .035), and Global (OR = 0.97, p = .066) READ-IT scores–corresponding to a lower text readability. In other words, a lower readability of web-based administrative information on induced abortion drove a less correct fruition of the abortion pathway, in contrast to the Italian law 194/1978. The effect of the Base READ-IT score was the highest, as suggested by the lower Odds Ratio. It suggests that a 1.0-point increase in the Base READ-IT score results in a -9% reduction in the odds of having a certified abortion (95% confidence interval: -1% to -16%).

**Table 3:** Multilevel logistic regression model.

Logistic models												
Certified abortion		OR	Std. Err.		OR	Std. Err.		OR	Std. Err.		OR	Std. Err.
	**Lexical READ-IT score**	**0,94** *	0,025	**Syntactic READ-IT score**	**0,95** *	0,023	**Base READ-IT score**	**0,91** *	0,040	**Global READ-IT score**	0,97	0,014
Age		**0,97** **	0,007		**0,97** **	0,007		**0,97** **	0,007		**0,97** **	0,007
Gestational age after 90 days vs. before		**0,14** ***	0,044		**0,14** ***	0,044		**0,14** ***	0,044		**0,14** ***	0,044
non-Italian vs. Italian nationality		1,12	0,109		1,12	0,110		1,12	0,110		1,12	0,110
High education level vs. low		0,98	0,108		0,98	0,108		0,98	0,108		0,98	0,108
Unemployed vs. employed		**1,34** **	0,126		**1,34** **	0,126		**1,34** **	0,127		**1,34** **	0,127
Student vs. employed		**2,10** ***	0,370		**2,10** ***	0,370		**2,11** ***	0,371		**2,10** ***	0,370
Married vs. single		0,88	0,094		0,88	0,094		0,88	0,094		0,88	0,094
Other marital status vs. single		1,17	0,215		1,17	0,215		1,17	0,215		1,17	0,216
Medical vs. surgical abortion		1,08	0,099		1,08	0,098		1,08	0,098		1,08	0,098
Number of previous children		**1,12** *	0,053		**1,12** *	0,053		**1,12** *	0,053		**1,12** *	0,053
Number of previous induced abortions		1,07	0,052		1,07	0,052		1,07	0,052		1,07	0,052
Waiting times		1,00	0,001		1,00	0,001		1,00	0,001		1,00	0,001
Hospital-level variance		1,99	0,604		2,04	0,620		1,96	0,593		2,11	0,649

Significance levels

* p < .05

** p < .01

*** p < .001 (bold values represent statistically significant Odds Ratios).

he Stata command *melogit* was used to perform multilevel logistic models; the highest-level grouping variable was the provider hospital (26 groups).

OR: Odds ratio; SE: Standard Error; Nr.: Number.

Other factors influencing the probability of having a certified abortion were very similar between the four models (Table 3). For instance, older women were less likely to obtain the certification provided by family counselling centres. Women terminating pregnancy after the first 90 days did not obtain any certification more frequently than women aborting within the first 90 days of gestation. The odds of having a certified abortion were higher among unemployed women and students than among employed women. Women who had previous children were more likely to have a certified abortion.

## Discussion

We found that regional abortion rates in Tuscany were 5.2 per 1,000 women, in line with the data provided by the Italian Health Ministry and the international literature [6, 7]. Previous studies have demonstrated practice variation in abortion rates [32–35]. However, this is the first study to quantify variation using the Systematic Component of Variation (SCV). Indeed, SCV calculation revealed low variation in abortion rates, but high variation in certified abortion rates. Therefore, we can state that, in Tuscany, variation in the proper fruition of the abortion pathway is greater than variation in access to abortion services. For this reason, we sought to explore some determinants of such variation.

First, we observed that the web-based texts analysed in this paper can be considered as easy to read when compared similar previous studies concerning other health services. For instance, Russo *et al*. found that the readability level of the top ten websites informing headache/migraine patients was low [36], and Kecojevic *et al*. showed that the main websites describing Preexposure Prophylaxis against HIV exceeded the reading skills of most U.S. adults [37]. In addition, two studies from Italy that explored the readability of web-based information on waiting times [13] and Covid-19 vaccination [38] reported much higher average READ-IT scores than those computed here. Secondly, we found that a higher readability of the web-based information on induced abortion provided by Tuscan institutional websites had positive impacts on the fruition of the care pathway. Indeed, weak communication due to poor harmonization and unclear provision of information can undermine efforts to effectively engage patients and guide them toward proper use of services.

In particularly, the Base READ-IT score was found to have a prominent effect. This confirms that the use of raw text features, such as sentence and word length, has a main impact on the level of linguistic complexity. Indeed, longer sentences are generally more grammatically complex than shorter ones; moreover, shorter words tend to be more frequent thus easier-to-read than longer words, which are less frequent and possibly specific of the domain of text thus being more difficult for layman people. However, as we have previously discussed, besides such raw text features there are other important factors making complex a sentence such as the lexicon exploited and its structure.

This study had two main findings: there is a certain level of variation in the utilization of abortion services among Tuscan health districts; and proper utilization of the abortion pathway is driven by higher readability of web-based administrative information. However, the main limitation of this study is that–even though we included in our statistical models several covariates–readability of web-based administrative information on the abortion pathway is only one determinant of variation that cannot fully explain the entire unwarranted variation in the abortion care pathway since many other factors that we did not consider in our study may impact variation.

Furthermore, we were not sure that the woman sought information about induced abortion from institutional websites, and we did not consider other sources from which the woman might have drawn such information. In addition, we assumed–without being certain–that those women that had an abortion in a TH sought information on the TH website itself, and not from another TH or LHA website. Other limits of this work are related to the data quality and availability. Administrative databases lack information on some patient features, such as family conditions, lifestyle, and income brackets. Moreover, potential coding error might have affected the correct identification of the type of abortion and of the counselling certification possession. Also, other factors potentially influencing the correct fruition of the abortion pathway at the micro- (patient-) and macro- (district-) levels might have not been considered.

However, the quality of health administrative databases of Tuscany is routinely checked by the Regional Health Information System Office, making such data reliable. Indeed, previous studies employing such data to explore practice variation have been published [39]. So, these data are well-validated, easily accessible, and allow gathering real-world information about the entire population of interest [40]. Therefore, despite its limitations, this is the first study using health administrative data to explore variation in abortion rates in Italy and to suggest the importance of web-based content readability for fostering the correct fruition of the abortion pathway.

This study has several implications. Low variation occurs in the access to abortion services in the Italian Region of Tuscany, but larger variation exists in the correct fruition of the abortion pathway. Such variation *may* be partially explained by a different complexity and readability of the web-based institutional information on the abortion pathway. However, further research is needed to assess all the determinants of variation and confirm the role of institutional text readability within the global context.

Nonetheless, the Web is increasingly becoming the main vehicle for information on access to the abortion care pathway [41]. Despite this, the literature shows that web-based information about the abortion pathway has difficult readability [14]. This paper is the first to explore the readability of web-based institutional texts on induced abortion in Italy using a validated tool for the Italian language. We analysed just six texts; however, these texts were obtained from the official websites of the three Local Health Authorities and the three Teaching Hospitals of Tuscany. Since abortion is a public health service, the public health institutions are responsible for providing clear and easily readable information on the proper fruition of the abortion pathway in their official websites, to ensure equity of access across their territories. This is even more true if–as our study suggests–the readability of the institutional websites’ contents on induced abortion drives variations in access to the care pathway.

## Conclusions

In conclusion, this study was the first to demonstrate variation in access to and use of the abortion care pathway in Italy and to analyse the readability of the content of institutional websites informing women on how to properly access the abortion care pathway. Our findings also suggest that this variation may in part be driven by the readability of the contents of institutional websites. So, given that the Web is becoming the main source of medical information–even regarding induced abortion–this study raises the need to monitor and improve the readability of institutional websites to ensure proper and also more equitable access to public abortion services.

## Supporting information

S1 Fig Abortion rates among under-25 and non-Italian women. (TIF)

S1 Table Details on data sources and variable selection. (DOCX)

S2 Table READ-IT scores for LHA and TH websites (and details on the READ-IT tool). (DOCX)

S3 Table Total and certified abortion rates for each health district. (DOCX)
