# Supplementary figures and images for: Higher readability of institutional websites drives the correct fruition of the abortion pathway: A cross-sectional study
Source: PLoS One. 2022 Nov 4;17(11):e0277342. doi: 10.1371/journal.pone.0277342 (PMC9635703; doi:10.1371/journal.pone.0277342)

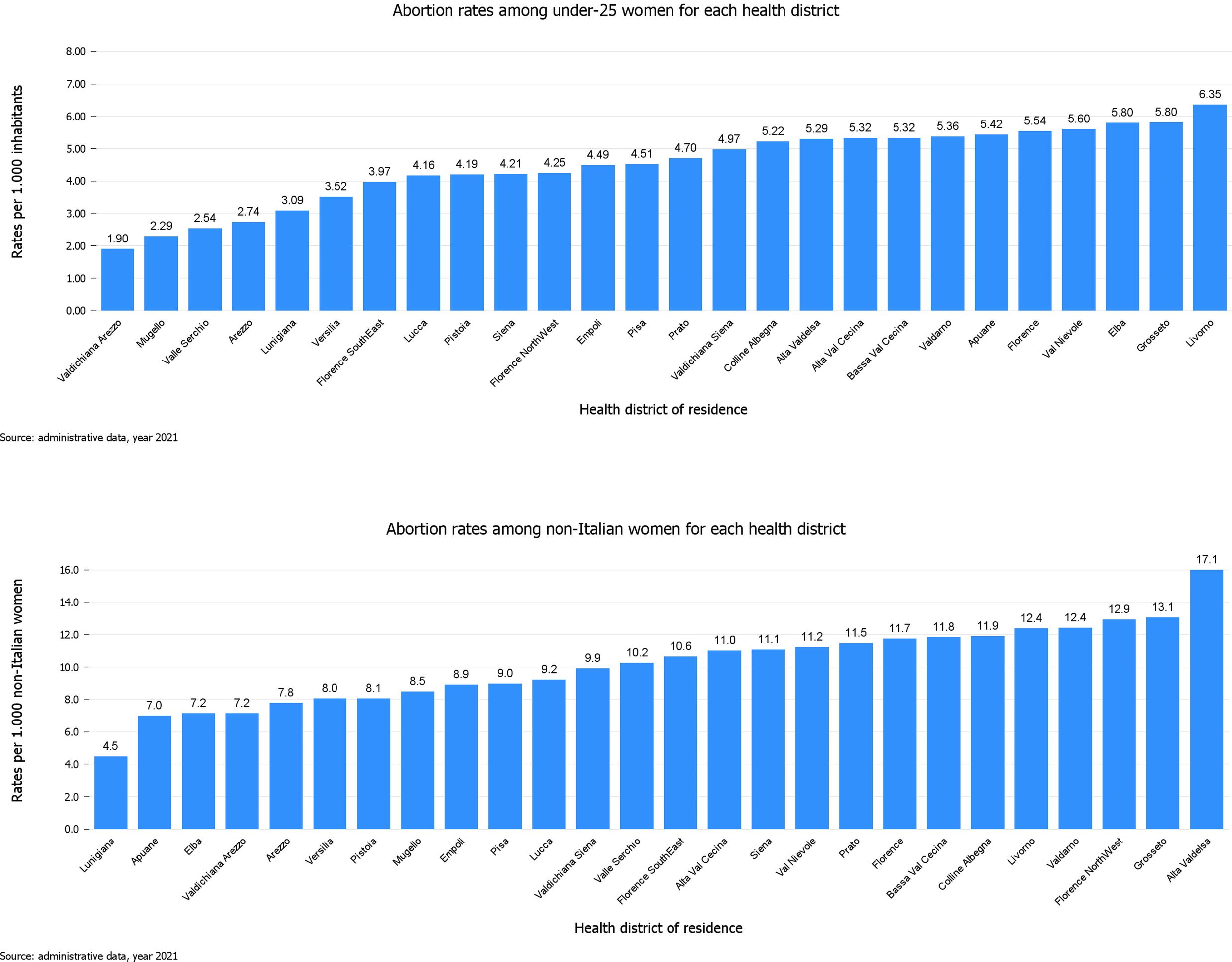

Supplement: S1 Fig — (TIF) [file pone.0277342.s001.tif]
