# Supplementary material for: Higher readability of institutional websites drives the correct fruition of the abortion pathway: A cross-sectional study
Source: PLoS One. 2022 Nov 4;17(11):e0277342. doi: 10.1371/journal.pone.0277342 (PMC9635703; doi:10.1371/journal.pone.0277342)
Supplement: S1 Table — (DOCX) [file pone.0277342.s002.docx]

**S1 Table.** Details on data sources and variable selection. We considered nationality (Italian or non-Italian), age, education level (low or high), employment status (employed, unemployed, or student), marital status (single, married, or other) and the type of abortion (medical or surgical). We also calculated for each woman the median waiting times from the request to the performance of the abortion. Finally, we included information on gestational age, number of previous children, and number of previous induced abortions.

|  |  |  |  |
| --- | --- | --- | --- |
| **Covariates** | | **Variable selection** | **Data source** |
| *Abortion-related covariates* | |  |  |
|  | Nationality (Italian or non-Italian) | variable "nationality" (namely, *cittu*) | Voluntary Termination of Pregnancy (VTP) Database |
|  | Age | variable "age" (namely, *eta*) |  |
|  | Education level (low or high) | variable "education level" (namely, *titstu*) |  |
|  | Type of abortion (medical or surgical) | variable "type of abortion" (namely, *tipo*) |  |
|  | Median waiting times from the request to the performance of the abortion | variables "request date" and "intervention date" (namely, *dat_rich* and *dataint*) |  |
|  | Employment status | variable "job" (namely, *condprof*) |  |
|  | Marital status | variable "marital status" (namely, s*tatciv*) |  |
|  | Gestational age | variable "gestational age" (namely, *etagest*) |  |
|  | Previous children | variable "children" (namely, *natvivi*) |  |
|  | Previous induced abortions | variable "voluntary termination of pregnancy" (namely, *ivg*) |  |
