# Supplementary material for: Higher readability of institutional websites drives the correct fruition of the abortion pathway: A cross-sectional study
Source: PLoS One. 2022 Nov 4;17(11):e0277342. doi: 10.1371/journal.pone.0277342 (PMC9635703; doi:10.1371/journal.pone.0277342)
Supplement: S2 Table — (DOCX) [file pone.0277342.s003.docx]

**S2 Table.** READ-IT scores for LHA and TH websites. The READ-IT tool relies on a given set of text features (i.e., lexical, morpho-syntactic, and syntactic characteristics of a document) and a training corpus (i.e., a collection of documents manually labelled with the level of linguistic complexity) and creates a statistical model using the feature statistics extracted from the training corpus. Such a statistical model is used in the assessment of readability of unseen texts based on the distribution of the aforementioned features, both at the whole document and at sentence level.

|  |  |  |  |  |
| --- | --- | --- | --- | --- |
| **Health authority** | **Lexical READ-IT score** | **Syntactic READ-IT score** | **Base READ-IT score** | **Global READ-IT score** |
| North-West LHA | 61,3 | 48,3 | 54,4 | 58,8 |
| Centre LHA | 69,4 | 56,8 | 58,2 | 73,7 |
| South-East LHA | 62,4 | 49,4 | 53,9 | 59,9 |
| Pisa TH | 66,6 | 55,8 | 70,8 | 55,8 |
| Florence TH | 61,7 | 35,0 | 47,7 | 35,0 |
| Siena TH | 59,5 | 54,2 | 60,6 | 54,2 |
|  |  |  |  |  |
