# Supplementary material for: Higher readability of institutional websites drives the correct fruition of the abortion pathway: A cross-sectional study
Source: PLoS One. 2022 Nov 4;17(11):e0277342. doi: 10.1371/journal.pone.0277342 (PMC9635703; doi:10.1371/journal.pone.0277342)
Supplement: S3 Table — (DOCX) [file pone.0277342.s004.docx]

**S3 Table.** Total and certified abortion rates for each health district of residence

|  |  |  |  |  |
| --- | --- | --- | --- | --- |
|  |  |  | Full study population | |
| **Health district** | **Health district code** | **Local Health Authority** | **Total number of abortions** | **% of certified abortions** |
| Lunigiana | 1011 | North-West | 29 | 89,7 |
| Apuane | 1012 | North-West | 160 | 80,0 |
| Valle Serchio | 1021 | North-West | 36 | 66,7 |
| Lucca | 1022 | North-West | 148 | 74,1 |
| Pistoia | 1031 | Centre | 157 | 63,7 |
| Val Nievole | 1032 | Centre | 131 | 75,6 |
| Prato | 1041 | Centre | 356 | 38,8 |
| Pisa | 1053 | North-West | 176 | 62,9 |
| Alta Val Cecina | 1054 | North-West | 133 | 68,4 |
| Livorno | 1062 | North-West | 185 | 64,9 |
| Elba | 1064 | North-West | 32 | 46,9 |
| Bassa Val Cecina | 1065 | North-West | 125 | 41,6 |
| Alta Valdelsa | 1071 | South-East | 89 | 43,7 |
| Siena | 1074 | South-East | 135 | 14,5 |
| Valdichiana Siena | 1075 | South-East | 84 | 35,7 |
| Valdichiana Arezzo | 1083 | South-East | 35 | 80,0 |
| Valdarno | 1085 | South-East | 112 | 75,9 |
| Arezzo | 1086 | South-East | 141 | 80,1 |
| Colline Albegna | 1092 | South-East | 39 | 64,1 |
| Grosseto | 1095 | South-East | 180 | 66,1 |
| Florence | 1101 | Centre | 476 | 46,0 |
| Florence NorthWest | 1102 | Centre | 251 | 35,9 |
| Florence SouthEast | 1103 | Centre | 180 | 52,2 |
| Mugello | 1104 | Centre | 45 | 51,1 |
| Empoli | 1113 | Centre | 249 | 41,8 |
| Versilia | 1121 | North-West | 140 | 40,0 |
